# Infective endocarditis causing recurrent cerebral infarction, cerebral hemorrhage and septic meningitis: A case report

**DOI:** 10.1097/MD.0000000000040749

**Published:** 2024-11-29

**Authors:** Huiliang Wang, Lingyan Fan, Chenxi Li, Haining Yu, Jilan Han, Yeliang Du, Guoping Xing

**Affiliations:** a School of Clinical Medicine, Shandong Second Medical University, Weifang, Shandong, China; b Department of Neurology, Weifang People’s Hospital, Weifang, Shandong, China.

**Keywords:** cerebral hemorrhage, cerebral infarction, infective endocarditis, septic meningitis

## Abstract

**Rationale:**

We reported a rare case of recurrent cerebral infarction, intracerebral hemorrhage, and purulent meningitis, culminating in the diagnosis of a young patient with infective endocarditis who had been treated in 3 hospitals for a long course of illness for 8 months prior to diagnosis. It aims to enhance clinicians’ understanding of the neurological complications caused by infective endocarditis.

**Patient concerns:**

A 25-year-old male, student, was hospitalized for an 8-hour history of speech impairment and drooling with dysphagia. Magnetic resonance imaging (MRI) showed massive cerebral infarction in the right frontotemporal and insular lobes, and the first diagnosis was “cerebral infarction.” Later, the patient developed recurrent cerebral infarction, intracerebral hemorrhage, and purulent meningitis.

**Diagnoses:**

Recurrent cerebral infarction, intracerebral hemorrhage, and purulent meningitis were confirmed to be caused by infective endocarditis.

**Interventions:**

The patient was treated with antiplatelet drugs such as aspirin and clopidogrel, mannitol to reduce intracranial pressure, and ceftriaxone and vancomycin to fight infection, and the patient’s condition improved.

**Outcomes:**

The patient was diagnosed with infective endocarditis after 8 months without a clear diagnosis, and the patient was finally diagnosed with infective endocarditis during the final follow-up.

**Lessons:**

Febrile patients should be aware of infective endocarditis, particularly if the fever is persistent of unknown origin or structural changes in the heart with neurologic lesions. Cardiogenic neurological diseases are relatively more severe, have a worse prognosis, and have a higher recurrence rate than primary neurological diseases, so early diagnosis and treatment are more urgently needed.

## 1. Introduction

Infective endocarditis (i.e.) is an acute or subacute disease of the endocardial valves or lining of the ventricular wall that occurs as a result of direct infection by bacteria, fungi, or other pathogenic microorganisms. Neurological complications are the most common and serious extracardiovascular complications of infective endocarditis. Approximately 25% of patients with infective endocarditis experience at least 1 neurological event, including cerebrovascular, infectious, or systemic disease.^[1]^ Early detection and treatment of infective endocarditis is particularly important to control neurological complications. A rare case of infective endocarditis in a young patient with recurrent cerebral infarction, cerebral hemorrhage, and purulent meningitis admitted to Weifang People’s Hospital has been reported. The patient was diagnosed at 3 hospitals with a delayed course of 8 months and had insidious cardiac symptoms but many extracardiovascular complications, which have not been reported in the literature. This case was reviewed, and the relevant literature was reviewed to improve clinicians’ understanding of infective endocarditis complicated by neurological disease.

## 2. Case report

**Figure 1.**
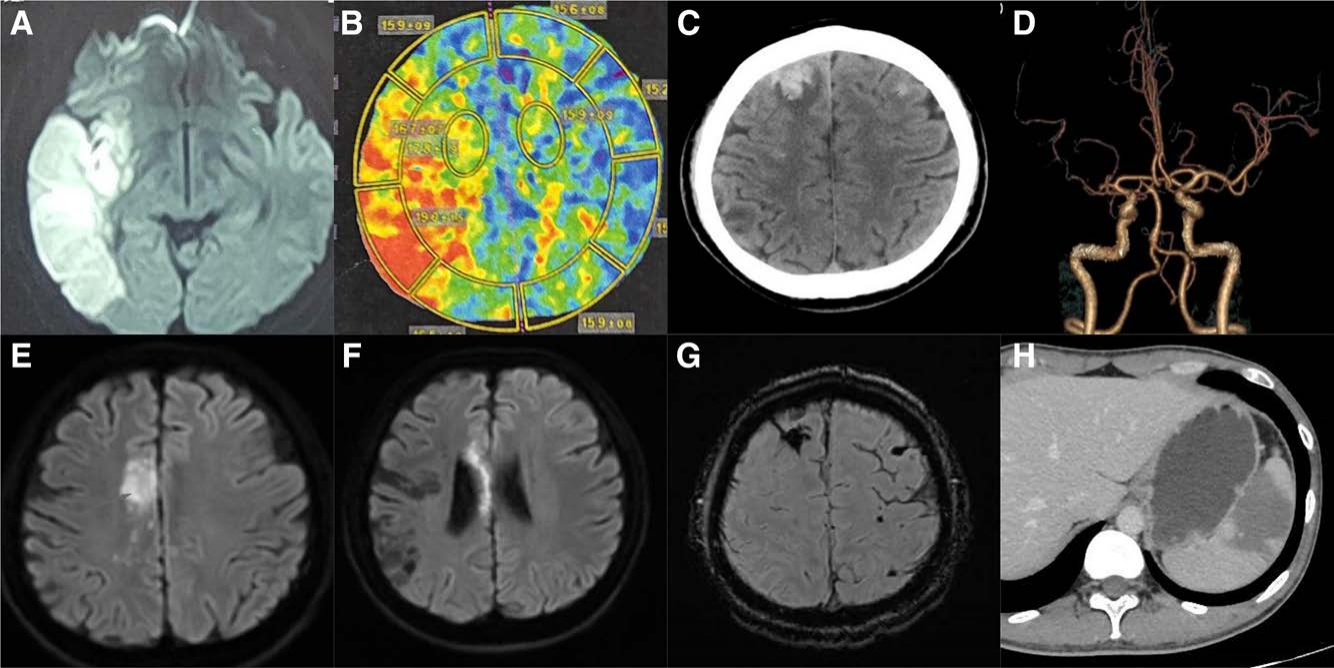
(A) Cranial MRI on June 22, 2021, showed: a large cerebral infarction in the right frontotemporal lobe and insula. (B) Cranial CTP on June 22, 2021, showed: severe stenosis of the proximal segment of M1 of the right middle cerebral artery nearly occluded, with sparse distal branching, ischemia, and hypoperfusion of the corresponding blood-supplying area, 62 mL of ischemic semidarked band, and large cerebral infarction in the right frontotemporal lobe. (C) Cranial CT on September 23, 2021, showed: patchy high-density shadows with clear margins were seen in bilateral frontal lobes, and low-density shadows were seen around them. (D) Cranial CTA on September 23, 2021, showed occlusion of the M1 segment of the right middle cerebral artery. (E) Cranial MRI on January 29, 2022, showed DWI sequences, and diffusely restricted signals were seen in the right frontal-parietal lobe. (F) Cranial MRI on January 29, 2022, showed DWI sequences, and a focally low signal was seen in the right corpus callosum. (G) Cranial SWI sequence on January 29, 2022, bilateral frontal-parietal lobe and part of the sulcus, right temporal lobe see low signal shadow. (H) Abdominal CT on February 12, 2022, showed splenic infarct foci. CT = computed tomography, CTA = computed tomography angiography, CTP = computed tomography perfusion, DWI = Diffusion-weighted imaging, MRI = magnetic resonance imaging, SWI = susceptibility weighted imaging.

The patient was a 25-year-old male and a student. He was admitted to an outpatient hospital on June 22, 2021, because of “unfavorable speech and, salivation with dysphagia for 8 hours.” On June 21, 2021, the patient experienced a headache after waking up in the morning, his temperature was measured at 38°C, and he was treated with self-administered ibuprofen. On June 22, the patient experienced salivation, poor speech, and weakness in chewing and swallowing, and was admitted to a hospital for treatment. A craniocerebral Magnetic resonance imaging (MRI) showed a large area of cerebral infarction in the right frontal-temporal lobe and insula lobe (Fig. 1A), and he was diagnosed with cerebral infarction. The patient was admitted to a hospital in Shanghai, China for treatment. Auxiliary examination: Transesophageal cardiac ultrasound showed that the foramen ovale was not closed (0.6 cm); Cranial computed tomography perfusion (CTP) showed that the proximal end of the right middle cerebral artery, section M1, was severely stenosed and nearly occluded, and the distal branches were sparse, with the corresponding blood-supplying areas suffering from ischemia and hypoperfusion. There was a large area of right frontal-temporal lobe cerebral infarction (Fig. 1B); the blood culture did not detect any causative organisms; There were no abnormalities in blood and urine, brain natriuretic peptide (BNP) and thyroid function. The patient was treated with aspirin and clopidogrel for antiplatelet therapy, atorvastatin for lipid stabilization and plaque stabilization, mannitol and glyceryl fructose for dehydration to lower cranial pressure, baclofen to alleviate intractable hiccups and ceftriaxone. The patient’s body temperature was normal and the symptoms gradually improved, with residual intractable hiccups, which did not affect limb movement and speech. The patient was discharged on June 30, 2021 and continued to receive oral aspirin, clopidogrel, and atorvastatin after discharge.

Because the patient had a patent foramen ovale during the cardiac ultrasound examination during the last hospitalization, he planned to undergo foramen ovale closure on July 30, 2021 in a hospital in Jinan, Shandong Province, but no patent foramen ovale was found during the operation. Ancillary examinations: Cardiac ultrasound showed EF56%, bilobed aortic valve, left ventricular enlargement, mitral regurgitation (mild to moderate), and tricuspid regurgitation (mild). The patient presented with intractable ergogenic symptoms resulting from a cerebral infarction. During hospitalization, he was administered: aspirin and clopidogrel for antiplatelet therapy, rivaroxaban for anticoagulation, atorvastatin for lipid regulation and plaque stabilization, cefoperazone sulbactam for anti-infection, baclofen to reduce intractable hiccups and acupuncture. The patient was discharged from the hospital on August 1, 2021 after the above treatments for a significant reduction in hiccups and no obvious positive signs on examination, and continued oral treatment with clopidogrel, rivaroxaban, atorvastatin, baclofen and trimethoprim after discharge. The symptoms of hiccups gradually recovered after discharge.

On September 20, 2021, the patient visited our hospital because of a headache for 3 days, and cranial computed tomography (CT) showed patchy high-density shadows in the bilateral frontal lobes, with clear edges and low-density shadows in the surrounding area (Fig. 1C). He was diagnosed with a cerebral hemorrhage and was admitted to the neurosurgery department for treatment. No obvious positive signs were observed on admission. Auxiliary examination: Cranial CTA showed occlusion of the right middle cerebral artery (Fig. 1D). During the hospitalization, the body temperature fluctuated between 36°C to 38°C. Tranexamic acid, mannitol, white-browed snake venom hemagglutinin, atorvastatin calcium tablets, debenone tablets, and baclofen tablets were administered, and clopidogrel and rivaroxaban were discontinued because of craniocerebral CT showing bilateral frontal lobe hemorrhage. Craniocerebral CTA was repeated on October 1, 2021, showing that the absorption of the hematoma was satisfactory, the patient’s headache symptoms improved, he was discharged from the hospital on October 4, 2021, and there were no obvious positive signs on discharge examinations. The patient continued to take atorvastatin calcium tablets, debenone tablets, and baclofen tablets treatment.

On January 26, 2022, the patient was admitted to our hospital because of “left limb immobility for 10 hours.” On January 26, 2022, the patient woke up and left limb immobility was found, which manifested as clumsiness in the left upper limb, dropping of objects, and dragging of the left lower limb, accompanied by headache, which was a persistent swelling and pain in the right frontal region, detected by cranial brain MRI in a local hospital. Improvement in cranial brain MRI showed new infarct foci in the right frontoparietal lobe and corpus callosum. The patient’s symptoms gradually worsened, and he was admitted to our hospital for treatment of “cerebral infarction.” Admission examination: temperature 37.5°C (temperature increased to 38.5°C on the following day); blood pressure: 106/66mmHg; mental clarity; dysphoria; fluent and tangential language; partial cooperative examination. The cranial nerve (−), left upper limb muscle strength grade 3, left lower limb muscle strength grade 0, right limb muscle strength grade 5, left limb muscle tone was low, left ataxia examination was uncooperative, bilateral pathological signs were not elicited, neck resistance and, 3 transverse fingers of the jaw and chest were present. NIHSS score 6. Ancillary examinations: cranial MR scan showed: new infarct foci in the right frontoparietal lobe and corpus callosum; soft foci and laminar necrosis in the right radiocoronal area and frontoparietal temporal lobe; abnormal low signal in the frontoparietal lobe and part of the sulcus, and in the right temporal lobe (Figs. 1E and F); cervical magnetic resonance angiography(MRA), and cranial MRV did not show any abnormality; cranial MRA: partial occlusion of the M1 segment of the right middle cerebral artery, and narrowing of the right anterior cerebral artery; cranial susceptibility weighted imaging(SWI): Bilateral frontal-parietal lobes and part of the sulcus, right temporal lobe, abnormal low signal shadow (Fig. 1G); cardiac ultrasound: small amount of regurgitation in the aortic valve, small amount of regurgitation in the mitral valve, small amount of regurgitation in the tricuspid valve; abdominal CT: infarcted foci of the spleen (Fig. 1H); blood: RBC 3.95 × 10^12^/L, HGB 110 g/L, GRAN 85.9%; blood sedimentation rate of 72 mm/h; CRP 45 mg/L Antinuclear antibody spectrum: ANA (cytoplasmic granular) 1:100; Calcitonin: 0.103ug/L; A-function, anti-O + rheumatoid factor, antineutrophil cytoplasmic antibody, lupus anticoagulant, quantitative antinuclear antibody (ANA), anti-beta2 glycoprotein I antibody (quantitative), immunoglobulin IgG, IgA, IgM, IgE, complement C3, C4, Hepatitis B, Syphilis, acquired immune deficiency syndrome(AIDS) and other antibodies, TSPOT, Buraphycin and other antibodies. and other antibodies, TSPOT, brucellosis-related antibodies, lipids, blood glucose, cardiac enzyme profile + troponin, electrolytes, liver and kidney function, coagulation routine + D-dimer, and blood homocysteine levels were not abnormal. The patient was admitted to the hospital and administered aspirin for antiplatelet aggregation, rosuvastatin calcium tablets for lipid regulation and plaque stabilization, edaravone for scavenging oxygen-free radicals, and butylphthalide to promote collateral circulation. On January 28, 2022, the patient experienced nausea, vomiting, headache, and fever with a body temperature of 38.4°C. The fever was accompanied by chills, indicating the possibility of bacterial infection, especially bacteremia. Ceftriaxone was given to treat the infection, and blood culture tests were also completed. The lumbar puncture was performed on January 29, 2022 to measure the cerebrospinal fluid pressure of 140mmH_2_ O, cerebrospinal fluid routine: white blood cell count 0.899 × 10^9^/L; cerebrospinal fluid biochemistry: protein 1074.0mg/L, glucose 2.18mmol/L (immediate blood glucose) Cerebrospinal fluid biochemistry: protein 1074.0mg/L, glucose 2.18mmol/L (instant glucose 6.7mmol/L), chloride 114.9mmol/L. No bacterial or fungal growth was observed in the complete cerebrospinal fluid culture. Diagnosed as “purulent meningitis” based on the patient’s symptoms and cerebrospinal fluid biochemical examination results. Then, on the basis of the original ceftriaxone treatment, vancomycin 1.0 q12h combined with anti-infective therapy was immediately added, and methylprednisolone treatment was given. On February 1, 2022, the patient’s blood culture revealed Streptococcus constellation. On February 6, 2022, a lumbar puncture was performed again to measure the cerebrospinal fluid pressure of 90 mmH_2_ O, cerebrospinal fluid routine: leukocyte count of 0.015 × 10^9^/L, cerebrospinal fluid protein 212.0 mg/L, Cerebrospinal fluid metagenomic next-generation sequencing: Moraxella (G-bacillus). No bacterial or fungal growth was observed during cerebrospinal fluid culture again.

The patient’s symptoms gradually improved. On February 10, 2022, the patient was rechecked again for lumbar puncture cerebrospinal fluid pressure of 65 mmH_2_ O, cerebrospinal fluid routine: leukocyte counts 0.020 × 10^9^/L, cerebrospinal fluid protein 121.0 mg/L. On February 20, 2022, he was rechecked again for lumbar puncture cerebrospinal fluid pressure of 65 mmH_2_ O, cerebrospinal fluid routine: cell counts 0.010 × 10^9^/L, cerebrospinal fluid protein 186.0 mg/L, vancomycin, and ceftriaxone were discontinued, and hormone dosage was reduced to 40 mg QD. The patient was discharged from the hospital on February 23, 2022. Discharge examination revealed mental clarity, fluent and tangential speech, bilateral nasolabial groove symmetry, left upper limb muscle strength grade 5, left lower limb proximal muscle strength grade 5-, distal muscle strength grade 4, normal muscle tone, right limb muscle strength grade 5, normal bilateral deep and superficial sensory examination, left lower limb tendon reflexes (++++), bilateral baroreflexes were not elicited. The patient was discharged from the hospital and continued to take oral prednisone tablets (40 mg QD) that were gradually tapered off.

On March 10, 2022, the patient underwent a cardiac ultrasound examination at an external hospital after discharge. The results showed bicuspid aortic valve malformation, aortic valve vegetation formation, and perforation with severe aortic regurgitation involving the root of the anterior leaflet of the mitral valve. Further aortic valve replacement surgery was performed in cardiac surgery, and the patient’s condition remained stable after the surgery. We conducted a 2-year follow-up on the patient, and his symptoms were stable, allowing him to work and live normally.

## 3. Discussion

The patient’s clinical presentation consisted of recurrent episodes of cerebral infarction, cerebral hemorrhage, and purulent meningitis combined with splenic infarction over an 8-month period, which led to a final diagnosis of infective endocarditis. The annual incidence of infective endocarditis (i.e.) is 3 to 10 cases/100,000 people.^[2]^ Rheumatic heart disease, degenerative valve disease, diabetes mellitus, cancer, intravenous drug use, and congenital heart disease are common risk factors for infective endocarditis.^[3]^ Staphylococci, streptococci, and enterococci account for 80% to 90% of infective cases, with streptococci accounting for 36% of cases.^[4]^ Neurological complications of infective endocarditis can be categorized as cerebrovascular (ischemic stroke, transient ischemic attack, intracranial hemorrhage, and mycotic aneurysm), infectious (meningitis, brain abscess, vertebral osteomyelitis and encephalopathy involving the spinal cord and/or nerve roots), and systemic (encephalopathy and seizure) diseases.^[1,5]^ This young stroke patient had a rare combination of neurologic complications such as cerebral infarction, cerebral hemorrhage, septic meningitis and splenic infarction, with a delayed course of more than 8 months before the final diagnosis was made for a variety of complex reasons.

Patients present with stroke onset in youth. Risk factors for stroke in youth reported in the literature include hypertension, dyslipidemia, diabetes mellitus, tobacco use, obesity, moyamoya disease, sickle cell disease, rheumatic heart disease, AIDS, tuberculosis, and arterial dissection.^[6]^ García-Cabrera et al reported that ischemic stroke is the most common neurological manifestation of infective endocarditis, with a prevalence of 37% to 83%.^[1]^ The pathogenic mechanism is the embolization of cerebral vessels by friable thrombi or dislodged bacterial emboli containing large amounts of hyaline from infectious organisms, or infectious vasculitis. Sustained inflammation mediated by pathogens, T cells, macrophages, and proinflammatory cytokines leads to intimal thickening, vascular occlusion, or vasospasm, and ultimately to cerebral infarction or ischemia. The risk of septic embolism is primarily influenced by the size and activity of the vegetations, Staphylococcus aureus infection, and mitral valve involvement.^[7–9]^ The patient had recurrent cerebral infarction, and no vegetations were found in several cardiac ultrasound examinations in the 8-month medical history, indicating that the results of routine transthoracic echocardiography were not abnormal, and the possibility of cardiogenic stroke could not be ruled out. The patient’s prolonged undiagnosed condition may be due to the weak pathogenicity and low virulence of the pathogenic bacteria, as well as intermittent use of antibiotics, which puts the body in a long-term chronic bacterial infection state. The patient had recurrent intermittent fever during the course of the illness, and the fever accompanied by chills after this hospitalization was consistent with the clinical manifestations of a chronic recurrent infection. In the event of weak resistance, the bacteria proliferate and then shed the bacterial organisms and embolize the cerebral blood vessels, which results in repeated cerebral infarctions in the patient. Splenic infarction is similar to cerebral infarction, in which the splenic artery is blocked by septic emboli, resulting in the infarction of the corresponding arterial blood-supplying area. Septic emboli not only cause embolism but may also lead to arteritis and subsequent cerebral vascular rupture and hemorrhage.

Hemorrhagic stroke accounts for nearly 20% of cerebrovascular complications of infective endocarditis, and 15% of these cerebrovascular lesions are hemorrhagic transformations of ischemic lesions.^[5]^ In patients with i.e., 1-third of cerebral hemorrhages are hemorrhagic transformations of ischemic infarcts caused by the progression of septic emboli, and the mechanism responsible for cerebral hemorrhage may be the rupture of an infected arteritis due to a septic thrombus.^[10]^ However, the absence of ischemic infarction at the right frontoparietal and corpus callosum hemorrhage sites, in this case, was not consistent with the hemorrhagic transformation of ischemic infarction, and the simultaneous presence of several microhemorrhages foci at nonhemorrhagic sites in the patient’s cranial SWI sequences suggests that there was the extensive involvement of vascular lesions or systemic disease secondary to the hemorrhage. In a prospective study by Klein I et al^[11]^ in which 57% of 130 patients with i.e. included were observed to have disseminated microhemorrhages in the brain, microhemorrhages may be due to subacute microangiopathy caused by immune vasculitis or embolic processes in the vasculature. Mycotic aneurysms causing cerebral hemorrhage are relatively rare, accounting for <10% of neurological complications of i.e., and are essentially inflammatory lesions caused by septic emboli that disrupt the intima of the vessel to form a mycotic aneurysm, which ruptures as a sudden subarachnoid hemorrhage or intracerebral hemorrhage.^[12]^ Mycotic aneurysms are usually pike-shaped, thin-walled, multiple, and distally located. Angiography can be performed to determine the presence of mycotic aneurysms and prevent recurrent cerebral hemorrhage in the brain.^[1,13,14]^ In this case, the patient was not diagnosed with i.e. at the time of the cerebral hemorrhage and was treated with antithrombotic drugs secondary to the hemorrhage without cerebral angiography to determine whether the cerebral hemorrhage was due to a mycotic aneurysm. The patient not only had complications of repeated cerebral infarction and cerebral hemorrhage but also had a rare combination of purulent meningitis.

The patient presented with headache, nausea, vomiting, and fever of 38°C to 39°C on January 28, 2022, and was diagnosed with “purulent meningitis” on the basis of clinical presentation and cerebrospinal fluid findings. Central nervous system (CNS) infection is an uncommon neurological manifestation of infective endocarditis, present in 1% to 6% of patients with infective endocarditis.^[1]^ Septic emboli composed primarily of Staphylococcus aureus (S. aureus) are the most likely mechanical cause.^[5]^ There are no reports on the pathophysiological mechanism of septic meningitis, and it is speculated that the possible mechanism is that after the bacteria contained in the septic embolus invade the subarachnoid space through the vascular wall, due to the lack of effective immune defense, the bacteria multiply in large quantities leading to the occurrence of septic meningitis. At the same time, the antigenic components of the bacterial wall and certain cytokines mediating inflammatory reactions stimulate the vascular endothelial cells, inducing a series of inflammatory pathological changes in the soft meninges.

We further analyzed the cause of infective endocarditis in this patient, and there was a correlation with the bicuspid aortic valve. Sara Couto Pereira et al,^[15]^ published a meta-analysis of 5351 patients with a bicuspid aortic valve, in which 184 patients with bicuspid aortic valves developed infective endocarditis during the follow-up period, with an incidence rate of 48.13/10,000 patients/year (95% CI 22.24–74.02), which, after adjusting for estimation, was associated with a 12-fold increase in risk compared with the general population (RR: 12.03, 95% CI 5.45–26.54). The risk factors for infective endocarditis in this young patient may arise from the bicuspid aortic valve and the specific mechanism by which different blood flow patterns of the bicuspid aortic valve result in damage to the endocardium at sites subjected to turbulent washout, and platelet and fibrinogen deposition promotes the seeding of blood-borne bacteria or fungi, leads to the production of vegetations.^[16]^

The modified Dukes criteria^[17]^ indicate that positive serial blood cultures and growths on cardiac ultrasound are the 2 main criteria for diagnosing infective endocarditis. Unfortunately, only 1 blood culture was performed during the patient’s hospitalization, and the blood culture turned out to be Streptococcus constellation. Unfortunately, no serial blood cultures were performed, which was further confirmed. In addition, because transthoracic ultrasonography did not reveal valvular growths, but we considered infective endocarditis and discussed it with cardiologists, and finally failed to confirm the diagnosis of infective endocarditis due to the lack of evidence from transthoracic ultrasound, the examination of transesophageal ultrasound with clearer and more accurate imaging was not further improved, so the main diagnostic criteria for imaging infective endocarditis were missing. These decisions are the main reason preventing us from being able to diagnose infective endocarditis while a patient is in the hospital.

In addition, the patient was not clearly diagnosed for more than 8 months, and the cause of this series of diseases was infective endocarditis only at the later follow-up. Reflecting on the reasons for the early omission of diagnosis of infective endocarditis in this patient, it may be that: the patient was in the early stage of infective endocarditis, the bacterial pathogenicity was weak, and the degree of valve lesions in the early stage of the disease was not easy to be detected; The site of the lesions was hidden and not easily detect; The diagnosis of infective endocarditis is also related to the level of the cardiac ultrasound operator; The intermittent application of antibiotics by the patient inhibited the bacterial proliferation and masked the manifestations of the disease and other factors.

## 4. Conclusions

Infectious endocarditis is difficult to diagnose in the early stages due to the complex and diverse pathogenic bacteria and the extensive use of antibiotics. According to the modified Dukes criteria,^[17]^ the patient’s cardiac ultrasound examination showed bicuspid aortic valve malformation and aortic valve vegetation formation. This is consistent with 1 of the main indicators for diagnosing infective endocarditis. The patient in this case has recurrent fever ≥ 38°C; Repeated occurrence of cerebral artery embolism leading to cerebral infarction; and occurrence of intracranial hemorrhage; The blood culture result shows Streptococcus constellation, which meets the 3 secondary diagnostic criteria, thus confirming the diagnosis of infective endocarditis. In clinical practice, patients have fever, especially persistent fever of unknown cause ≥ 38°C or cardiac structural changes, as well as cerebral infarction, cerebral hemorrhage, and nervous system infectious diseases.^[18]^ Attention should be paid to the microbiological results of continuous blood culture, echocardiographic examination results, Osler nodules, rheumatoid factor, and other indicators. Early diagnosis and treatment of infective endocarditis can reduce related complications and improve patients’ quality of life.

## Author contributions

**Conceptualization:** Huiliang Wang.

**Data curation:** Huiliang Wang.

**Formal analysis:** Huiliang Wang.

**Investigation:** Lingyan Fan.

**Methodology:** Chenxi Li.

**Project administration:** Huiliang Wang.

**Resources:** Haining Yu.

**Software:** Jilan Han.

**Supervision:** Guoping Xing.

**Validation:** Huiliang Wang.

**Visualization:** Yeliang Du.

**Writing – original draft:** Huiliang Wang.

**Writing – review & editing:** Guoping Xing.
